# Educators’ perspectives for modernizing dental teaching competencies at Prince Sattam Bin Abdulaziz University-Saudi Arabia: a needs assessment study

**DOI:** 10.3389/fmed.2025.1669301

**Published:** 2025-11-10

**Authors:** Ali Robaian, Nasser Raqe Alqhtani, Abdullah Saad Alqahtani, Abdullah Alshehri, Khaled M. Alzahrani, Tarek Ahmed Soliman

**Affiliations:** 1 Department of Conservative Dental Sciences, College of Dentistry, Prince Sattam Bin Abdulaziz University, Al-Kharj, Saudi Arabia; 2 Department of Oral and Maxillofacial Surgery and Diagnostic Sciences, College of Dentistry, Prince Sattam Bin Abdulaziz University, Al-Kharj, Saudi Arabia; 3 Department of Preventive Dental Sciences, College of Dentistry, Prince Sattam Bin Abdulaziz University, Al-Kharj, Saudi Arabia; 4 Department of Prosthetic Dental Sciences, College of Dentistry, Prince Sattam Bin Abdulaziz University, Al-Kharj, Saudi Arabia

**Keywords:** needs assessment, education, teaching, dentistry, professional competence, Saudi Arabia

## Abstract

**Introduction:**

Learners are more likely to adopt new behaviors if interventions are planned according to needs assessment. Consequently, studies are required to analyze the perceptions of oral healthcare professionals prior to the initiation of a training program. This cross-sectional study aimed to evaluate the perceptions of oral healthcare professionals at Prince Sattam Ben Abdulaziz University regarding the current trainings for teaching competencies to identify and address critical areas for improvement.

**Methods:**

An online questionnaire was developed and distributed to oral healthcare professionals through institutional email using a secure Google Forms link. The questionnaire comprised of five sections: demographic information, feedback on previous training development programs related to teaching skills, and feedback on ‘self-rated performance’ versus ‘perceived importance’ on didactic and clinical teaching competencies. The feedback about the training delivery method was also included in the questionnaire. A Delphi validation method and Cronbach’s alpha were employed to evaluate the questionnaire’s validity and reliability. Upon collecting all responses, descriptive statistics were conducted to analyze the frequency distribution of the data.

**Results:**

Sixty-six participants completed the survey, achieving a response rate of 75%. In terms of the overall feedback on previous training concerning teaching competencies, participants assessed these as poor or fair, good, and very good to excellent at rates of 34–40%, 27–33%, and 27–32%, respectively. A significant difference (*p* < 0.05) was observed between self-rated performance and perceived importance in four out of seven items related to course design competencies, three out of seven items related to course delivery competencies, and three out of six items related to student assessment competencies. Face-to-face interactive group sessions training (85.71%) is the preferred method for delivery of the training sessions.

**Conclusion:**

Within the limitations of this study, the needs assessment identified areas of interest for teaching competencies that need to be prioritized at the College of Dentistry at Prince Sattam Bin Abdulaziz University. Priority is given to the assistant professors and teaching assistants for developing twelve teaching competencies. On the other hand, professors and associate professors identified seven competencies to be updated for their respective knowledge.

## 1 Introduction

Saudi Arabia is undergoing a paradigm shift across all sectors of the country. Education and healthcare are among the top priorities in the 2030 development plan. Saudi Vision 2030 aims to modernize and enhance the quality of education, aligning it with global standards to equip Saudi students for future challenges. In alignment with the Saudi Vision 2030 agenda, dentistry colleges must enhance training in teaching methods and educational theory, as this is an essential step for curriculum modernization. Training programs in teaching competencies are principally vital in adapting oral healthcare professionals to their changing roles in initiating and setting directions for curricular changes (1–3).

Prince Sattam Ben Abdulaziz University (PSAU) is one of the modern universities in the Kingdom of Saudi Arabia that was established in 2009. A faculty development program (FDP) is a structured activity that improves an individual’s knowledge and competencies in academically important areas for meeting future development demands (4, 5). FDPs prepare the faculty to adapt to rapid changes in healthcare delivery, clinical practice, and medical education. It is also important for promoting effective educational innovation and ensuring that oral healthcare professionals are well-trained (6). Faculty members represent the most important resources in higher education institutions; therefore, FDP should serve as a resource that supports their individual goals (7, 8).

The needs assessment represent the initial phase in the development of an effective training program. It uses a systematic approach for data collection and analysis to assess individuals’ current competencies, needs, gaps between current and desired conditions, and the most effective scheduling and delivery methods for training interventions (9). Learners tend to adopt new behaviors if interventions are planned according to needs assessment surveys (10). A variety of tools and techniques, including questionnaires, focus groups, interviews, and Delphi procedures, can be employed to conduct needs assessments for continuing medical education in diverse contexts (11). Adkoli et al. (12) emphasized the necessity of recognizing disparities between “perceived importance” and “self-rated performance” as essential indications for prioritization. Their findings emphasized the necessity of a comprehensive faculty development plan that integrates both departmental and institutional initiatives. Similarly, Wallin et al. (13) stated that understanding gaps between what is important and the level of competence aids in the strategic focus of professional development initiatives, allowing decision-makers to maximize the effectiveness of constrained resources for educational enhancement.

The College of Dentistry faces a significant challenge as its current curriculum is built on a conventional model that necessitates conversion into a competency-based framework aligned with international dental education standards. This shift underlines the importance of comprehensive faculty development activities to provide educators with the abilities required for the successful implementation of competency-based education. Secondly, there is an opportunity for collaboration with the Saudi Commission for Health Specialties to establish a postgraduate training program entitled “Saudi Board in Family Dental Medicine.,” which is built on CanMEDS framework. Consequently, effective training in teaching competencies should be designed to meet the specific needs of institutions, departments, and oral healthcare professionals. This will ensure that graduating students and postgraduate healthcare professionals have the knowledge, competencies, and attributes to prevent and manage oral diseases effectively, collaborate across healthcare disciplines to improve health, and fulfill their professional and personal responsibilities. Furthermore, the rationale of an effective training program is to commit to the strategic goals of the university and college, as well as Saudi Vision 2030.

To our knowledge, there is a lack of studies on the development of advanced training in dental teaching competencies based on reliable integral demands in Saudi Arabia. Studies are required to analyze the perceptions of oral healthcare professionals prior to the initiation of a training program. It is essential to prioritize the training activities based on the disparity between expected competencies and actual performance. Furthermore, evaluating faculty perceptions regarding professional development demand is essential for aligning training initiatives with curricular modernization efforts and maintaining sustainable academic quality. Accordingly, this study investigated the perceptions of oral healthcare professionals at Prince Sattam Ben Abdulaziz University regarding the current trainings in teaching competencies to identify and address critical areas for improvement.

## 2 Methodology

### 2.1 Study design and ethical approval

This study utilized a cross-sectional survey design. The authors are a group of educational specialists in the dentistry and dental education fields, who created an online faculty development needs assessment (FDNs) tool to assess the perceptions of oral healthcare professionals at the College of Dentistry-Prince Sattam Bin Abdulaziz University (PSAU) about current training programs and highlight their critical areas for improvement concerning didactic and clinical teaching competencies. All procedures performed in this study adhered to the principles outlined in the Helsinki Declaration. Incomplete responses were excluded from the data analyses. This study was conducted following the CHERRIES guideline for electronic surveys. The Standing Committee of Bioethics Research at PSAU approved the study and assigned approval number SCBR-348/2024. The study was conducted with the support of a faculty development initiative funded by the Deanship of Scientific Research at PSAU (project number 2024/03/29306).

### 2.2 Study settings

The need assessment was designed following the guidelines for survey construction, including questionnaire length and design, as well as previous literature reviews of FDNs and institutional needs. The questionnaire items were designed using a close-ended format to enable quantitative analysis. Three medical education experts evaluated the content and face validity using a modified Delphi method. Based on their feedback, the questionnaire was modified and finalized. The content and face validity ratios were 0.83 and 0.92, respectively. The questionnaire underwent testing with 30 participants at the College of Dentistry (PSAU) to evaluate the questions’ clarity and measure the time needed for completion. The time required for completion of the questionnaire was 9 min. Cronbach’s alpha was used to assess reliability within each competency domain, yielding values of 0.81 for course design, 0.84 for course delivery, and 0.88 for student assessment. A highly satisfactory reliable level is indicated by a Cronbach’s alpha of 0.8 or higher (11).

### 2.3 Participants and recruitment

The questionnaire’s target population is full-time faculty members at the College of Dentistry-PSAU. Surveys were distributed to all participants’ official email addresses via a secure link on the Professional version of Survey Monkey. Participants received a pre-notification invitation and informed consent letter 1 week before the survey distribution. Participants consented to including their data in an aggregate report during the survey. The faculty scheme included a total of 87 faculty members. The sample size was calculated using a 90% confidence interval, a 60% response distribution, and a margin of error of ± 5%. The sample size required, calculated with Raosoft® (Raosoft, Inc., Seattle, Washington, USA), was 66.

### 2.4 Questionnaire

The survey consisted of 29 items and took no longer than 10 min to complete (Supplementary material). The questionnaire was carried out between October and November 2024. The introductory part of the questionnaire asked for responses from the respondents regarding their demographic characteristics(e.g., Gender, Nationality, Academic position, dental specialty, kind of teaching activity they practice, and their teaching experience) (6 questions). After the demographic questions, the questionnaire had 4 main sections. The first part (2 questions) sought to evaluate the participants’ feedback on previous training related to teaching competencies and indicate the frequency of their participation. Five Likert scales were used; [1] = Poor, [2] Fair, [3] = Good, [4] = Very good, [5] = Excellent. The second part of the questionnaire assessed participants’ feedback on ‘self-rated performance’ versus ‘perceived importance’ on twenty didactic and clinical teaching competencies related to course design (7 questions), course delivery (7 questions), and student assessment methods (6 questions). Five Likert scales were used for self-rated performance; [1] = little, [2] average, [3] = good, [4] = Approaching mastery, [5] = Mastery/could teach others. Three Likert scales were used for perceived importance competencies; [1] = Not at all important [2] = Moderately important [3] = Extremely important. The third part (1 question) assessed the preferred method for conducting the training activities.

### 2.5 Statistical analyses

Statistical analyses were performed using the statistical package for Social Science (SPSS) version 27 (IBM, Armonk, USA). Descriptive statistics were used for the frequency distribution of all the responses. The descriptive statistics were used to analyze respondent characteristics. The authors dichotomized scores for the self-performance rating (lower: knowledge = 1, 2, 3 versus high: knowledge = 4, 5) and priority scores (lower: priority = 1, 2 versus high: priority = 3). The Chi-Square test was used to analyze and identify the academic rank differences in self-performance and their priority.

## 3 Results

### 3.1 Characteristics of participants

**Figure 1:**
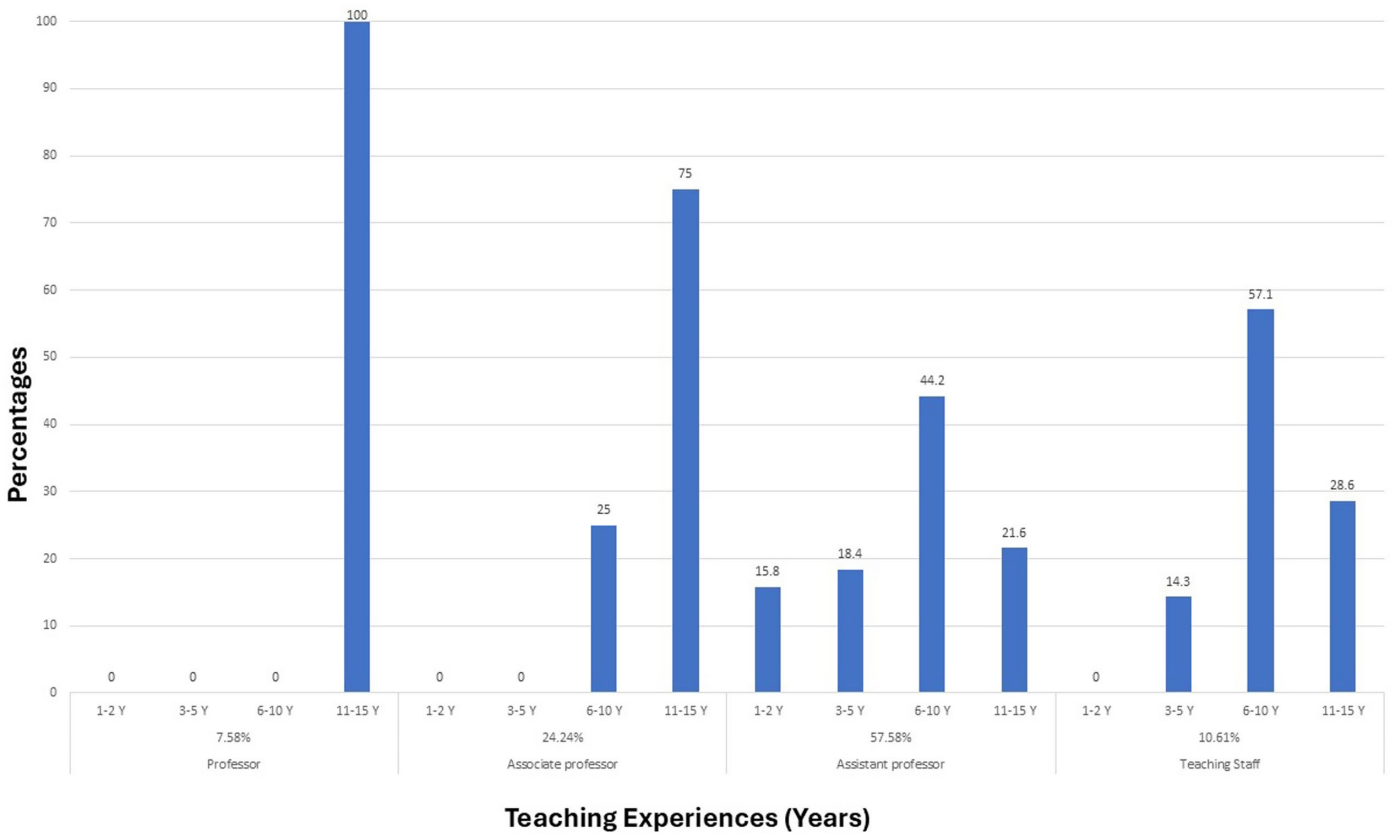
Teaching experience for each academic rank in the college.

Sixty-six participants completed the survey, achieving a response rate of 75.8% of the whole faculty in the college. According to the participants’ academic positions, the majority were assistant professors (57.58%), followed by associate professors (24.24%), teaching assistants staff (10.61%), and professors (7.58%). Gender-wise distribution revealed (78.79%) male and (21.21%) female responders. In terms of teaching experience, the majority of participants had taught for a duration of 11–15 years (40.91%), followed by those with 6–10 years (37.88%) of teaching experience. Some had 3–5 years of teaching experience (12.12%) and fewer had 1–2 years of teaching experience (9.09%). The specialty department revealed 22.73% from department A, 22.73% from department B, 27.27% from department C, 15.15% from department D, and 12.12% from department E (Table 1). Figure 1 shows the teaching experience for each academic rank in the college.

**Table 1:** Characteristics of participants (*N* = 66).

Variables		Frequency	Percentages (%)
Gender	Male	52	78.79
	Female	14	21.21
Academic position	Professor	5	7.58
	Associate professor	16	24.24
	Assistant professor	38	57.58
	Teaching Assistant staff	7	10.61
Department specialty	A	15	22.73
	B	15	22.73
	C	18	27.27
	D	10	15.15
	E	8	12.12
Teaching experience (years)	1–2	6	9.09
	3–5	8	12.12
	6–10	25	37.88
	11–15	27	40.91

### 3.2 Faculty’s rating of the current dental teaching competencies

In terms of the overall feedback on previous training concerning teaching competencies, participants assessed these as poor or fair, good, and very good to excellent at rates of 34–40%, 27–33%, and 27–32%, respectively. The professor rated the training strategy (1.80 ± 0.32) and venue (1.80 ± 0.31) the lowest scores in the current training activities. Associate professors rated the topics (2.19 ± 0.78) and trainers (2.13 ± 0.76) with the lowest scores in the current training activities. The assistant professors rated all current training activities with the lowest scores ranging from 1.71 ± 0.03 to 1.8 ± 0.09. The overall participants’ response across 5 levels of participation (never, rarely, sometimes, often, and frequently) was 7.58, 39.39, 36.37, 9.09, and 7.57%, respectively. 80% of the professors and 75% of associate professors attend ≥ 4 workshops per year. Regarding assistant professors (57.14%) and assistant staff (71.4) ranged from never to rarely attending training workshops. There were statistically significant differences (*p* > 0.05) between participants’ satisfaction and level of participation in the training programs.

### 3.3 Participants’ self-performance rating versus priorities in dental teaching competencies

Any item in the Knowledge and Priority columns with a percentage of more than 40% was rated as a high need and high priority (Tables 2–4).

**Table 2:** Participants’ self-rated performance and perceived importance (%) in course design competencies, categorized by academic rank (*n* = 66).

Competencies	Rating	Academic positions (percentages)				
		Professor (*n* = 5)	Assoc. prof (*n* = 16)	Assist. prof (*n* = 38)	Teaching assistant (*n* = 7)	Total % (*n* = 66)
C1. Developing instructional goals and objectives	LK	20%	37.5%	26.3%	71.4%	38.8%
	HP	20%	43.7%	30.3%	100%	48.5%
	*p* value	0.92	0.18	0.67	0.045*	0.55
C2. Design Dental Course specification	LK	0%	37.5%	36.4%	51.4%	31.3%
	HP	20%	30.8%	47.4%	85.7%	45.9%
	*p* value	0.78	0.32	0.52	0.039*	0.74
C3. Appropriate selection of teaching methods for Course goals	LK	0%	31.3%	36.9%	57.1%	31.3%
	HP	20%	43.7%	47.3%	81.4%	48.1%
	*p* value	0.79	0.62	0.34	0.041*	0.063
C4. Developing blueprint	LK	0%	37.5%	42.1%	85.7%	41.3%
	HP	40%	43.8%	78.9%	100%	65.7%
	*p* value	0.05*	0.63	0.86	0.98	0.05*
C5. Design problem-based teaching activity	LK	40%	43.7%	66.7%	57.1%	51.8%
	HP	80%	56.3%	69.7%	100%	76.5%
	*p* value	0.045	0.97	0.89	0.035*	0.042*
C6. Designing OSCE/OSPE stations	LK	40%	56.3%	63.6%	85.7%	61.4%
	HP	100%	82.5%	91.5%	100%	93.5%
	*p* value	0.034*	0.05*	0.041*	0.83	0.024*
C7. Designing team-based learning activity	LK	40%	50%	65.8%	100%	63.9%
	HP	100%	62.5%	69.6%	100%	83.1%
	*p* value	0.037*	0.56	0.65	0.95	0.037*

LK, Lower Knowledge; HP, Higher Priority. *p value is statistically significant.

**Table 3:** Participants’ self-rated performance and perceived importance (%) in course delivery competencies, categorized by academic rank (*n* = 66).

Competencies	Rating	Academic Positions (Percentages)				
		Professor (*n* = 5)	Assoc. prof (*n* = 16)	Assist. prof (n = 38)	Teaching Assistant (*n* = 7)	Total % (*n* = 66)
D1. Lecture presentation skills	LK	0%	37.5%	36.9%	28.5%	28.3%
	HP	20%	31.2%	34.2%	42.8%	32.1%
	*p* value	0.88	0.59	0.79	0.098	0.34
D2. Teaching using various “smart” technologies	LK	40%	31.3%	52.6%	57.1%	45.2%
	HP	80%	37.5%	73,4%	71.4%	65.6%
	*p* value	0.05*	0.742	0.032*	0.022*	0.042*
D3. Developing online teaching materials	LK	40%	43.7%	44.7%	42.8%	40.8%
	HP	80%	68.7%	67.3%	28.6%	61.2%
	*p* value	0.05*	0.013*	0.015*	0.089	0.034*
D4. Encouraging student participation in classes	LK	20%	31.3%	36.8%	28.5%	29.2%
	HP	40%	25%	47.4%	42.8%	42.4%
	*p* value	0.78	0.61	0.093	0.083	0.16
D5. Mentoring students	LK	20%	37.4%	42.1%	42.8%	35.6%
	HP	40%	43.7%	76.5%	100%	65.1%
	*p* value	0.77	0.72	0.004*	<0.001*	0.034*
D6. Facilitating small-group discussion	LK	20%	31.3%	36.8%	85.7%	45.4%
	HP	20%	43.7%	47.4%	28.6%	34.9%
	*p* value	0.99	0.88	0.63	0.019*	0.086
D7. Teaching strategy in a large classroom	LK	0%	37.4%	52.6%	85.7%	43.9%
	HP	20%	43.8%	26.3%	28.6%	29.6%
	*p* value	0.88	0.43	0.003*	<0.001*	0.129

LK, Lower Knowledge; HP, Higher. *p value is statistically significant.

**Table 4:** Participants’ self-rated performance and perceived importance (%) in student assessment competencies, categorized by academic rank (*n* = 66).

Competencies	Rating	Academic Positions (Percentages)				
		Professor (*n* = 5)	Assoc. prof (*n* = 16)	Assist. prof (*n* = 38)	Teaching Assistant (*n* = 7)	Total % (*n* = 66)
S1. Identifying and assisting students experiencing difficulty	LK	0%	37.5%	42.1%	42.8%	34.5%
	HP	20%	43.8%	34.2	57.1%	45.9%
	*p* value	0.096	0.234	0.751	0.081	0.231
S2. Multiple source feedback (360 assessment method)	LK	40%	56.3%	55.3%	57.1%	52.2%
	HP	100%	81.3%	60.5%	71.4%	78.3%
	*p* value	< 0.001*	0.003	0.642	0.064	0.034*
S3. Assessment using (MCQs)	LK	0%	31.3%	42.1%	42.9%	29.1%
	HP	20%	43.8%	60.5%	28.6%	38.3%
	*p* value	0.098	0.56	0.097	0.061	0.193
S4. Different Assessment methods in clinical settings such as (DOPS), and (Mini-CEX).	LK	40%	68.8%	60.5%	42.9%	52.9
	HP	100%	81.3%	47.3%	57.1%	71.4
	*p* value	< 0.001*	0.056	0.193	0.084	0.0246*
S5. Developing Educational portfolio	LK	20%	37.5%	68.4%	57.1%	45.8
	HP	60%	43.8%	50%	14.3%	42.1
	*p* value	0.0043*	0.075	0.065	0.004*	0.842
S6. Assessing the professional behavior of students	LK	20%	62.5%	50%	42.9%	43.8
	HP	80%	75%	63.2%	57.2%	68.9
	*p* value	< 0.001*	0.094	0.086	0.086	0.023*

LK, Lower Knowledge; HK, Higher Knowledge; LP, Lower priority; HP, Higher Priority. *p value is statistically significant.

#### 3.3.1 Course design competencies

Overall, a statistically significant difference (*p* < 0.05) was observed in four of the seven teaching competency items (C4, C5, C6, and C7). Table 2 indicates that 41.3, 51.8, 61.4, and 63.9% of the faculty assessed their knowledge as low in the areas of developing blueprints, designing problem-based teaching, designing OSCE/OSPE stations, and designing team-based teaching activities, respectively. On the other hand, the faculty rated these teaching competencies as high priorities, between 65.7 and 93.5%. With a closer look at the data, the professors and associate professors identified three high-priority competencies (C5, C6, and C7), assistant professors identified four competencies (C4, C5, C6, and C7), and teaching assistants identified all seven competencies as high-priority competencies based on knowledge and priorities percentages greater than 40%.

#### 3.3.2 Course delivery competencies

A statistically significant difference (*p* < 0.05) was observed in three of the seven teaching competency items (D2, D3, and D5). Table 3 indicates that 45.2, 40.8, and 35.6% of the faculty rated their knowledge as low in the areas of teaching using smart technologies, developing online teaching materials, and mentoring students, respectively. Faculty who identified these activities as high-priority needs ranged from 65.6–76.4%. Regarding course delivery competencies, the professors identified two high-priority competencies (D2 and D3), the associate professor identified one high-priority competency (D3), the assistant professors identified three competencies (D2, D3, and D5), and the teaching assistants identified two competencies (D2 and D5)as high-priority competencies, based on knowledge and priorities percentages greater than 40%.

#### 3.3.3 Student assessment competencies

Overall, a statistically significant difference (*p* < 0.05) was observed in three of the six student assessment competency items (S2, S4, and S6). Table 4 indicates that 52.2, 52.9, 61.4, and 43.8% of the faculty assessed their knowledge as low in the areas of multiple source feedback activities, different Assessment methods in clinical settings such as (DOPS, and Mini-CEX), and assessing the professional behavior of students. On the other hand, the faculty rated these teaching competencies as high priorities in these low-rated competencies between 68.9 and 78.3%. With a closer look at the data and based on knowledge and priorities percentages greater than 40%, the professors identified two high-priority competencies (S2, S4, and S6), assistant professors identified five competencies (S2, S3, S4, S5, and S6), and teaching assistants identified four high-priority competencies (S1, S2, S4, and S6).

### 3.4 Top high-priority competencies categorized by academic ranks

Based on knowledge and priorities percentages greater than 40%, Professors identified seven competencies as high-priority competencies: three in course design (C5, C6, and C7), two in course delivery (D2 and D3), and two in student assessment methods (S2 and S4). The associate professor identified eight competencies as high-priority competencies: four in course design (C4, C5, C6, and C7), one in course delivery (D3), and three in student assessment methods (S2, S4, and S6). The assistant professors identified twelve competencies as high-priority competencies: four in course design (C4, C5, C6, and C7), three in course delivery (D2, D3, and D5), and five in student assessment methods (S2, S3, S4, S5, and S6). The teaching assistant identified thirteen competencies: all seven-course design competencies, two course delivery (D2 and D5), and four in student assessment competencies (S1, S2, S4, and S6) (Table 5).

**Table 5:** Top high-priority competencies categorized by academic ranks.

Competencies	Academic positions			
	Prof.	Assoc. prof	Assist. prof	Teaching assistant
C1. Developing instructional goals and objectives				■
C2. Design Dental Course specification				■
C3. Appropriate selection of teaching methods for Course goals				■
C4. Developing blueprint			⁘	■
C5. Design problem-based teaching activity	●	✓	⁘	■
C6. Designing OSCE/OSPE stations	●	✓	⁘	■
C7. Designing team-based learning activity	●	✓	⁘	■
D1. Lecture presentation skills				
D2. Teaching using various “smart” technologies	●		⁘	■
D3. Developing online teaching materials	●	✓	⁘	
D4. Encouraging student participation in classes				
D5. Mentoring students			⁘	■
D6. Facilitating small-group discussion				
D7. Teaching strategy in a large classroom				
S1. Identifying and assisting students experiencing difficulty				■
S2. Different methods for student feedback	●	✓	⁘	■
S3. Assessment using (MCQs)			⁘	
S4. Different Assessment methods in clinical settings such as (DOPS), and (Mini-CEX).	●	✓	⁘	■
S5. Developing Educational portfolio			⁘	
S6. Assessing the professional behavior of students		✓	⁘	■

The symbol indicates that this competency represents a developmental need for this academic rank.

### 3.5 Participants’ preferences for training activities delivery

Face-to-face interactive group sessions training (85.71%) are the preferred methods for delivery of the training sessions.

## 4 Discussion

The significant changes in Saudi Arabia’s healthcare system and educational priorities, as highlighted in its 2030 vision, have created an urgent demand for effective professional development in teaching competencies at the College of Dentistry-PSAU. Previous studies highlighted the critical importance of customized training programs in preparing healthcare professionals for educational roles, providing benefits for healthcare professionals, students, and the community (14–16). This will enhance the educational environment for learners and improve their academic performance. Consequently, this may result in a shift in instructional beliefs and practices within the Saudi educational community (17, 18).

Needs assessment helps in situation analysis and setting priorities for establishing a faculty development program to ensure quality improvement in education. This study aimed to evaluate the perceptions at the College of Dentistry-PSAU regarding the current training programs and highlight their critical areas for improvement regarding teaching competencies. However, in some areas, faculty sometimes prioritized items higher while their knowledge was low, and vice versa (19). The judging behavior of the faculty may differ. Furthermore, sometimes when faculty need specific skills, they do not think they are high-priority (19, 20). Accordingly, This study aimed to prioritize the training activities based on the disparity between expected competencies and actual performance. For this reason, any item in the Knowledge and Priority areas in this study with a percentage of more than 40% was rated as a high priority (19, 20).

Prince Sattam University provides a diverse array of training activities for its staff and employees, organized according to an annual schedule distributed before the commencement of the academic year. The assistant professors rated all current training activities with the lowest scores ranging from 1.71 ± 0.03 to 1.8 ± 0.09. This may be due to the inappropriateness of the training activities’ timing for them. Additionally, participation in these training activities is not mandatory for the staff member. Although this study indicated no statistically significant differences (*p* > 0.5) between participants’ satisfaction and their level of participation in the training programs, it is worth mentioning that 57.14% of assistant professors ranged from never to rarely attending training workshops. Furthermore, previous research showed that the research on behavioral changes showed that most effective methods are face-to-face interactive training sessions (14, 15, 19).

It is observed that professional development needs vary according to the academic rank. The findings indicate that assistant professors and teaching assistants exhibit a greater need for teaching compared to professors and associate professors, which can be interpreted as that they are more open to learning. These findings are consistent with Khan et al. (21) indicated that junior faculty tend to exhibit greater motivation for participating in structured professional development as they work to establish their teaching identities. Wilkerson et al. (22) and Steinert et al. (23) reported that early-career faculty place greater importance on pedagogical training compared to senior faculty, who typically depend on their accumulated experience rather than formal training. In contrast, in the current study, most professors and associate professors serve as clinical consultants at the Saudi Commission for Health Specialties and possess Training-of-Trainers (TOT) credentials, which may explain their significantly lower reported needs in specific competences.

There was a significant difference (*p* < 0.05) in four of seven items concerning course design competency (C4, C5, C6, and C7), three of seven items regarding course delivery competency (D2, D3, and D5), and three of six items related to student assessment competency (S2, S4, and S6). Nonetheless, all teaching competencies for faculty members across different academic ranks in the current study were prioritized based on low knowledge and high priority percentages exceeding 40%. This could be attributed to, sometimes, the faculty judging their knowledge to be low and their priorities on these items to be high. Conversely, at other times, faculty may judge their knowledge to be high and their priorities on these items to be high also (12, 19). Adkoli et al. (12) reported similar findings, indicating that self-assessment among health professions educators frequently reveals disparities between perceived competence and perceived importance, thereby identifying areas for focused professional development.

Designing problem-based learning (PBL), team-based learning (TBL), and OSCE/OSPE station competencies were identified as high-priority competencies. Furthermore, competencies related to student feedback, clinical student assessment, and professional behavior assessment were highly rated. These competencies represent the foundational elements for adopting a competency-based education (CBE) approach. These findings are consistent with previous studies (24, 25) that emphasized the necessity of faculty training in modern assessment methods and learner-centered strategies for a successful transition to CBE. Furthermore, the College of Dentistry at PSAU is currently preparing for a transition toward a competency-based curriculum, reinforcing the faculty’s prioritization on these competencies.

The use of different “smart” technologies and the development of online teaching materials were identified as a high-priority competency. This finding aligns with previous studies (26, 27) that reported enhanced awareness among faculty regarding the significance of digital literacy and online teaching in medical and dental education. Teaching assistants should receive special attention in this regard, since they play a crucial role in content development and facilitating online sessions (28). Online classes continue to serve as a vital backup strategy at the College of Dentistry at PSAU, particularly during emergencies.

The current study findings indicated that the face-to-face interactive sessions are the preferred method for faculty. This indicates that faculty favor active training that includes the application and practice of gained knowledge. These findings align with a prior study indicating that faculty prefer professional development activities in a workshop format (29).

The College of Dentistry at PSAU is currently preparing to undergo a paradigm shift from a traditional to a competency-based approach. There is a reciprocal relationship between new curricula and faculty development. Preparing faculty is a necessary adjunct to facilitate the design, implementation, and evaluation of new curricula. Additionally, faculty development may drive change to a new curriculum by fostering a change in attitudes, improving knowledge, or enhancing skills. Accordingly, these customized training activities must be compulsory for each academic rank since faculty members play a significant role in teaching as they are the center for ensuring student learning. This study has limitations; the data was collected solely from the College of Dentistry at PSAU-Saudi Arabia. The findings primarily reflect the institution where the study was conducted. The samples may not be representative of the entire faculty in two departments as the response rate was low in these two departments. Additionally, relying on one method of data collection (an online survey). Therefore, future prospective studies should employ qualitative need assessment to know the in-depth attitudes of faculty regarding their training needs.

## 5 Conclusion

Within the limitations of this study, the needs assessment identified areas of interest for teaching competencies that need to be prioritized at the College of Dentistry at Prince Sattam Ben Abdulaziz University.

- Assistant professors demonstrated the greatest need for development, with twelve teaching competencies identified as high-priority areas for training.
- Professors and associate professors indicated the need to update and refine seven competencies, reflecting their respective knowledge and experience levels.
- These findings emphasize the need for structured, rank-specific faculty development initiatives designed to enhance teaching effectiveness and align academic competencies with the evolving requirements of competency-based dental education.

## Data Availability

The original contributions presented in the study are included in the article/Supplementary material, further inquiries can be directed to the corresponding author.
