# Supplementary material for: Educators’ perspectives for modernizing dental teaching competencies at Prince Sattam Bin Abdulaziz University-Saudi Arabia: a needs assessment study
Source: Front Med (Lausanne). 2025 Nov 10;12:1669301. doi: 10.3389/fmed.2025.1669301 (PMC12640949; doi:10.3389/fmed.2025.1669301)
Supplement: Supplementary file 1 [file Table_1.docx]

**Modernizing Dental Teaching Competences at Prince Sattam Ben Abdulaziz University Saudi Arabia: Needs Assessment Cross-Sectional Study**

**Section 1. Demographic Information**

The College of Dentistry at Prince Sattam Ben Abdulaziz (PSAU) is committed to ongoing faculty development and advancement. Please take a few minutes to complete this survey, which is part of a study to assess faculty development needs, related to dental teaching skills. Faculty administration will use the findings to build evidence-based faculty development programs based on real needs.

You consent to participate in this study by completing this questionnaire. We guarantee your privacy; no personal information will be shared. Participants gave permission for their data to be included in an aggregate report.

Your response is highly valuable.

Thank you in advance.

1. **Your Name ( Optional)**
2. **Gender:** Male Female
3. **Nationality:** Saudi non-Saudi
4. **Current Academic Rank:**

- Professor
- Associate Professor
- Assistant Professor
- Lecturer
- Teaching Staff

1. **What is Your Department Specialty?**

- Prosthodontics
- Oral and Maxillofacial Surgery and Diagnostic Sciences
- Conservative Dental Sciences
- Preventive Dentistry
- Orthodontics

1. **Teaching Experience (Years)**

- <2
- 3-5
- 6-10
- 11-15
- 16-20

**Section 2: Satisfaction with the current faculty development activities**

1. **How many faculty development activities did you attend in the last three years?**

- **Never**
- **Rarely 1-2 per year**
- **Sometimes (3-4 per year)**
- **Often (5-7 per year)**
- **Frequently ( (8 or more per year)**

1. **What is your level of satisfaction with the existing faculty development activity program?**

| **Variables** | **Poor** | **Fair** | **Good** | **Very good** | **Excellent** |
| --- | --- | --- | --- | --- | --- |
| **Topics** |  |  |  |  |  |
| **Trainers** |  |  |  |  |  |
| **Teaching methods** |  |  |  |  |  |
| **Venue** |  |  |  |  |  |
| **Schedule/time** |  |  |  |  |  |

**Section 3: Teaching Skills Self-rating and Priorities**

listed below are some skills and knowledge areas. First, assess your current skill/knowledge level using the following scale: Five Likert scales were used for self-rated performance;

[1] = little, [2] average, [3] = good, [4] = Approaching mastery, [5] = Mastery/could teach others.

**Then**

Assess the level of priority for the perceived importance competencies;

Three Likert scales for perceived importance competencies;

[1] = Not at all important [2] =Moderately important [3] =Extremely important.

| **Competencies** | **Category** | self-rated performance  **[1] = little [2] average [3] = good [4] = Approaching mastery,  [5] = Mastery/could teach others.** | Priority within your personal development  **[1] = Not at all important   [2] =Moderately important   [3] =Extremely important.** |
| --- | --- | --- | --- |
| **1. Developing instructional goals and objectives** | **Course  Design** |  |  |
| **2. Design Dental Course specification** |  |  |  |
| **3. Appropriate teaching methods for Course goals** |  |  |  |
| **4. Developing blueprint** |  |  |  |
| **5. Design problem-based teaching activity** |  |  |  |
| **6. Designing OSCE/OSPE stations** |  |  |  |
| **7. Designing team-based learning activity** |  |  |  |
| **8. Lecture presentation skills** | **Course  Delivery** |  |  |
| **9. Teaching using various “smart” technologies** |  |  |  |
| **10. Developing online teaching materials** |  |  |  |
| **11. Encouraging student participation in classes** |  |  |  |
| **12. Mentoring students** |  |  |  |
| **13. Facilitating small-group discussion** |  |  |  |
| **14. Teaching strategy in a large classroom** |  |  |  |

| **Competencies** | **Category** | self-rated performance  **[1] = little [2] average [3] = good [4] = Approaching mastery,  [5] = Mastery/could teach others.** | Priority within your personal development  **[1] = Not at all important   [2] =Moderately important   [3] =Extremely important.** |
| --- | --- | --- | --- |
| **15. Identifying and assisting students experiencing difﬁculty** | **Student assessment methods** |  |  |
| **16. Multiple source feedback (360 assessment method)** |  |  |  |
| **17. Assessment using (MCQs)** |  |  |  |
| **18. Different Assessment methods in clinical settings such as (DOPS), and (Mini-CEX).** |  |  |  |
| **19. Developing Educational portfolio** |  |  |  |
| **20. Assessing the professional behavior of students** |  |  |  |

**Section 4: Delivery Methods for Faculty Development Program**

**What is your preferred method for conducting faculty development activities?**

- Face-to-face interactive group training sessions
- Web-based modules and E-mail-based discussion forums
- individualized consultation
- Online webinars
